# Risk Factors to Persistent Pain Following Musculoskeletal Injuries: A Systematic Literature Review

**DOI:** 10.3390/ijerph19159318

**Published:** 2022-07-29

**Authors:** Othman Alkassabi, Lennard Voogt, Pamela Andrews, Ahmad Alhowimel, Jo Nijs, Hana Alsobayel

**Affiliations:** 1 Pain in Motion International Research Group, Department of Physiotherapy, Human Physiology and Anatomy, Faculty of Physical Education and Physiotherapy, Vrije Universiteit Brussel, 1050 Ixelles, Belgium; othman.yousef.alkassabi@vub.be (O.A.); l.p.voogt@hr.nl (L.V.); 2 Physiotrio, Riyadh 13213, Saudi Arabia; 3 Research Centre for Health Care Innovations, Rotterdam University of Applied Sciences, 3015 GG Rotterdam, The Netherlands; jo.nijs@vub.be; 4 Healthcare Improvement Scotland, Glasgow G1 2NP, UK; pamela.andrews2@nhs.scot; 5 Department of Health and Rehabilitation Science, Sattam Bin Abdulaziz University, Al-Kharj 16278, Saudi Arabia; aalhowimel@gmail.com; 6 Chronic Pain Rehabilitation, Department of Physical Medicine and Physiotherapy, University Hospital Brussels, 1090 Jette, Belgium; 7 Unit of Physiotherapy, Department of Health and Rehabilitation, Institute of Neuroscience and Physiology, Sahlgrenska Academy, University of Gothenburg, 405 30 Gothenburg, Sweden; 8 The Research Chair for Healthcare Innovation, Department of Rehabilitation Sciences, College of Applied Medical Sciences, King Saud University, Riyadh 11451, Saudi Arabia

**Keywords:** persistent pain, systematic review, musculoskeletal injury, chronic pain

## Abstract

Background: Musculoskeletal (MSK) injury is one of the major causes of persistent pain. Objective: This systematic literature review explored the factors that lead to persistent pain following a MSK injury in the general population, including athletes. Methods: A primary literature search of five electronic databases was performed to identify cohort, prospective, and longitudinal trials. Studies of adults who diagnosed with a MSK injury, such as sprains, strains or trauma, were included. Results: Eighteen studies involving 5372 participants were included in this review. Participants’ ages ranged from 18–95 years. Most of the included studies were of prospective longitudinal design. Participants had a variety of MSK injuries (traumatic and non-traumatic) causing persistent pain. Multiple factors were identified as influencing the development of persistent pain following a MSK injury, including high pain intensity at baseline, post-traumatic stress syndrome, presence of medical comorbidities, and fear of movement. Scarcity of existing literature and the heterogeneity of the studies made meta-analysis not possible. Conclusions: This systematic review highlighted factors that might help predict persistent pain and disability following MSK injury in the general population, including athletes. Identification of these factors may help clinicians and other health care providers prevent the development of persistent pain following a MSK injury.

## 1. Introduction

Musculoskeletal (MSK) pain conditions are very common and are one of the top 20 causes for years lived with disabilities globally [1]. MSK injuries are one of the major causes of persistent pain leading to disabilities and high disease burden [2]. Persistent pain after MSK injury is not only common in the general population but also in athletes, leading to disability and time lost from sports activities [3]. Persistent pain is defined as pain that persists for three to six months following onset, according to the International Association for the Study of Pain [4].

It has been reported that only a small percentage of people will be free of pain following MSK trauma [5]. For that reason, the development of persistent pain following MSK injuries in the general population has been the subject of a number of studies [5,6,7], with one study finding that up to 48% develop chronic pain after traumatic MSK injuries, and a combination of social and medical risk factors identified in the development of chronic pain [5].

In sport-related MSK injuries, a recent scoping review explored the psychological, social, and contextual factors across recovery stages following a sport-related knee injury, finding a broad spectrum of psychological, social, and contextual factors that influenced recovery [8]. It was suggested in this review that athletes who suffered a sport-related knee injury experienced fear/anxiety as well as other barriers to recovery, most predominantly at the return to sport. It was also suggested that psychological, social, and contextual factors influencing recovery were dynamic over the stages of recovery. Central sensitization and psychosocial variables have also been considered to be explanatory factors for persistent pain after MSK injury [9,10].

The limited success seen for the management of persistent pain following MSK injuries in the general population underscores the need for strategies to prevent the development of persistent pain. In order to do this, it is important to understand the factors that contribute to the transition from acute to chronic pain following MSK injury. Therefore, the current systematic review explored the factors that lead to persistent pain following acute MSK injury in the general population. This will be the first step towards focusing on preventing persistent pain and shifting the focus towards prevention of chronicity following musculoskeletal injuries. The study findings may help identify modifiable factors to help prevent chronicity following MSK injury. Additionally, the current systematic review investigated the intrinsic factors (i.e., anatomical and psychological) and extrinsic factors (i.e., social and environmental) that predict the transition from acute to persistent pain state in individuals following MSK injury.

## 2. Materials and Methods

The search strategy was developed in accordance with the Preferred Reporting Items for Systematic Reviews and Meta-analysis (PRISMA) guidelines [11].

### 2.1. Search Strategy

A primary literature search of five electronic databases was performed to extract data from prospective and retrospective cohort studies. The search strategy was prepared by an information specialist from the Erasmus Medical Centre in Rotterdam, The Netherlands. Electronic databases, including MEDLINE, the Cumulative Index to Nursing and Allied Health Literature (CINAHL), PubMed, ProQuest, and Web of Science, were searched from their inception to June 2020. The reference lists of eligible studies and relevant systematic reviews were screened for additional articles. In addition, experts in the field were contacted to identify unpublished studies and corresponding authors from the included articles were, when necessary, consulted in order to clarify any missing data.

### 2.2. Selection Criteria

To systematically select studies, inclusion and exclusion criteria were developed a priori and were applied in three stages. In stage one, identified studies were exported into Covidence, an online systematic review-management platform, where two investigators (O.A. and P.A.) independently reviewed the titles and abstracts against predefined criteria. In stage two, relevant full text articles were retrieved and independently reviewed by two investigators (O.A. and P.A.) to determine their eligibility. The final stage involved screening the included full text studies to exclude unrelated studies. In the event where agreement could not be reached, a third investigator (H.S.) was consulted.

The literature search was conducted using the following criteria: (1) population: adults who had sustained a MSK injury, (2) types of studies: observational studies (retrospective, prospective, cross-sectional, and longitudinal studies), and (3) outcome of interest: pain following injury. Reviews, case reports, and studies that examined the epidemiology, examination, and treatment were excluded. No restriction was placed on language or date of publication. Adults over 18 years who had been diagnosed with injuries such as sprains, strains, or trauma through impact or fall were included. Individuals who presented at baseline with chronic pain or pain as a result of surgery or non-MSK injury were excluded. Studies were included when they examined outcomes associated with the measurement of pain and the factors associated with the development of acute to chronic pain.

### 2.3. Data Extraction

A data-extraction form was developed for the purposes of this review. Data extraction included the following information: (1) study characteristics (authors, year of publication, and study design); (2) participant characteristics (number of participants at enrollment and follow-up, demographic information, and injury characteristics); (3) risk factors identified; (4) outcomes measured; (5) estimates of risk factors and persistent pain (e.g., odd radios (ORs)); and (6) authors’ conclusions. Kappa statistics were used to assess agreement between the two investigators on inclusion at each stage of the review.

### 2.4. Quality Assessment

The methodological quality of each included study was assessed using the Quality in Prognosis (QUIP) checklist, which comprises six important domains (i.e., participation, prognostic factor measurement, attrition, outcome measurement, confounding measurement, and analysis and reporting) for assessing validity and risk of bias in prognostic studies [12,13,14]. Therefore, the current systematic review used the QUIP checklist to assess risk of bias in the included studies. Two independent investigators (O.A. and P.A.) evaluated the included studies based on these criteria [13]. The checklist items were evaluated independently as either ‘Identified’ (1 point) or ‘Not identified’ (0 point) by investigators and then discussed to reach consensus. If an agreement between the two investigators could not be met, a third investigator (HS) was consulted. The points from the QUIP checklist were totaled, and studies were considered as having low risk of bias if they were found to be of high quality (score ≥ 17/22) and high risk of bias if they were found to be of low quality (score ≤ 16/22), with this near the 80% quality cut-off point [14].

## 3. Results

### 3.1. Study Selection and Characteristics

**Figure 1:**
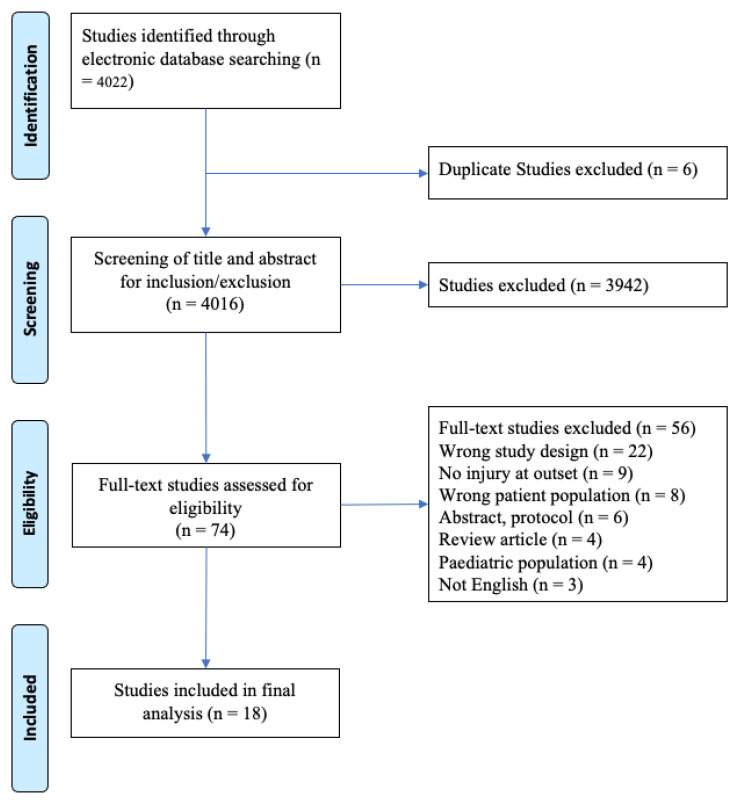
PRISMA flow diagram of the selected papers.

Out of 4022 identified studies, six duplicates were removed (Figure 1). Out of the remaining 4016 studies, 3942 studies were excluded during title and abstract screening. Out of 74 full-text studies, 56 studies did not meet the inclusion criteria. Finally, a total of 18 studies involving 5372 participants were included in this systematic review. Two independent investigators (O.A. and P.A.) examined the relevant articles and short-listed as per a priori risk-of-bias criteria.

Table 1 presents study characteristics, such as authors’ names, country of study, study design, and sample size. Included studies originated from Australia [15,16,17,18], United States [19,20,21], Canada [22,23], Denmark [24], Germany [25], The Netherlands [26,27], Sweden [28,29], Spain [30], and United Kingdom [31,32]. Participants’ ages ranged from 18–92 years. Most of the included studies used a prospective longitudinal design, only one study was cross-sectional in nature [15]. Participants in the included studies had various of MSK injuries (traumatic and non-traumatic MSK) that led to persistent pain. Minimum and maximum follow-up periods were one week [26] and five years [21], respectively. Most of the included studies measured pain intensity using a numerical rating scale (NRS) [16,17,24,26,27], while three studies used a visual analogue scale [18,30,31]. Sample sizes of the included studies ranged between 66 [21] and 1290 [18].

### 3.2. Study Quality (Risk of Bias)

**Figure 2:**
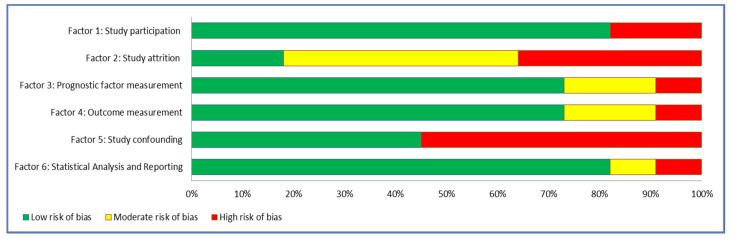
Assessor’s judgment about the risk of bias for each QUIIP factor across all included studies.

Table 2 presents the quality scores from each of the included trials. The risk-of-bias assessment, conducted by the two investigators, was found to be reliable (kappa coefficient = 0.85). Risk of bias was assessed separately for the six QUIP factors. More than 70% of included studies had a low risk of bias for most of the QUIP. Five studies had a high risk of bias for factor 2 and six studies for factor 5. Four studies had a moderate risk of bias for factor 2. Figure 2 presents the assessors’ judgments about the risk of bias for each QUIP factor presented as percentages across all included studies. Studies were considered with low quality if most criteria were not met, or significant flaws relating to key aspects of study design were evident. Five studies with low quality [15,20,25,30,31] were not included in the narrative synthesis of the results.

### 3.3. Risk Factors for Persistent Pain

Multiple risk factors for developing persistent pain following MSK injury were identified. However, due to the between-study heterogenicity and the limited number of studies examining each risk factor, it was not possible to run a meta-analysis of the results. Therefore, a narrative synthesis of results was conducted. Table 3 presents details of risk factors contributing to persistent pain following MSK injuries as identified through this systematic review.

Age was found to be a significant risk factor for developing chronic pain after MSK injury in a study by Pierik, IJzerman et al. [26].

Initial pain severity was reported as a risk factor for the development of chronic pain in three studies [16,17,19]. Pain at the time of discharge from hospital after traumatic MSK injury predicted the development of chronic pain in one study [28]. The severity of the MSK injury was found to predict the course of pain in MSK injuries [26].

One study reported that the presence of comorbidities predicted chronic pain after MSK injury [28]. In this study, comorbidities were defined as having three or more chronic medical conditions (e.g., diabetes, and hypertension). A low level of physical activity was reported to be a predictor of chronic pain in MSK injuries in two studies [21,28]. One study demonstrated that the level of education and eligibility for compensation following MSK injury may act as risk factors for the development of chronic pain [21].

The presence of post-traumatic stress disorder was shown to be a risk factor for developing chronic pain following MSK injury in one study [25]. One study reported that fear avoidance and catastrophizing may be risk factors for chronic pain [27]. In a sample of patients after distal radius fractures, Ref. [26] reported that depression was a significant risk factor for slowing recovery after the injury.

## 4. Discussion

This systematic review explored the factors that contribute to persistent pain following acute MSK injury in the general population. Many of the included studies identified persistent pain following MSK injury. Similarly, Rosenbloom et al. [2] reviewed 11 studies and they concluded high prevalence of persistent pain following traumatic musculoskeletal injury. The results highlighted several modifiable and non-modifiable risk factors leading to chronicity in patients who experienced a MSK injury. The results of this study contribute to the body of knowledge on factors leading to persistent pain following MSK injuries that will help guiding prevention strategies to reduce the burden of these conditions.

Comparing our results to previous research, many of the studies included in this review identified persistent pain following MSK injury [15,16,17,18,19,20,21,22,23,24,25,26,27,28,29,30,31,32,33]. Personal factors such as age, which is considered to be a non-modifiable factor, have reported association with persistent pain. Most of the studies included in the current review reported that the prevalence of persistent pain following MSK injury was more common in those of middle age. In contrast, a previous review identified older age as one of the predicting factors for persistent pain following MSK injury [2]. The reason for this is not clear because heterogeneity in the study design and methodology precludes direct comparison. For instance, four included studies in this review had reported persistent pain following musculoskeletal injuries in more than 60% of female patients. Likewise, a previous study reported high risk of chronic pain following trauma in female patients [34].

Our finding that persistent pain after MSK injury was associated with a group of modifiable factors, including high intensity of pain, pain-catastrophizing, fear-avoidance beliefs, and post-traumatic stress symptoms, is similar to that of a previous review which identified predictive factors including initial pain, anxiety and depression, fear-avoidance, and patient perception for persistent pain [2]. Another study reported high risk of persistent pain in patient with high levels of general anxiety and post-traumatic stress symptoms [22]. Moreover, several studies had reported a positive relationship between post-traumatic stress symptoms and chronic pain [35,36,37,38].

Other modifiable risk factors identified in the current review for developing persistent pain after MSK injury included total abbreviated injury score, initial pain severity, and initial pain control attitudes, which concur with previous studies. Similarly, other studies reported several risk factors of pain progression in traumatic patients [3,39]. Some of the factors are present at the time of admission (e.g., injury pattern and type, anxiety and depression), some are present during hospitalization (e.g., pain intensity, type of surgery, treatment strategies, and hospital-stay duration), while others are present at the time of discharge (e.g., anxiety and depression, post-traumatic stress symptoms, and pain catastrophizing) [2,3,40,41].

None of the studies included in this review investigated or reported risk factors for persistent pain following a sport MSK injury. Therefore, there is a need for more research to understand the transition from acute to chronic pain following sports MSK injury, preferentially applying a broad biopsychosocial perspective and sport-related perspectives for identifying potential risk factors. This will inform health and medical programs at all levels (preventive, primary, secondary, and tertiary) in order to reduce disability following MSK injuries.

The current review had several strengths as well as limitations. Strengths included the screening of five electronic databases by two independent investigators, the search strategy which was prepared by a specialized and independent information specialist, the risk-of-bias assessment performed by two independent investigators, the high interrater reliability of the risk-of-bias assessment, the compliance with the international standards for conducting and reporting systematic literature reviews (i.e., the PRISMA guidelines) and the detailed and thorough data processing. Hence, all efforts were undertaken to optimize the internal and external validity of the study findings, yet some study limitations should be mentioned. First, heterogeneity in the included studies prevented the ability to directly compare various factors causing persistent pain following MSK injury. Second, most of the included studies in this review were cross-sectional in nature, preventing the ability to conduct a cause-and-effect analysis. Finally, a relatively small number of studies (n = 11) were included in this review due to the scarcity of studies that fulfil the inclusion criteria. Therefore, more studies using larger and more homogenous study populations are warranted to further identify various predictors of persistent pain following MSK injury in the adult general population.

## 5. Conclusions

There are multiple factors causing persistent pain following MSK injury in the general population. These factors include high intensity of pain, pain-catastrophizing, fear-avoidance beliefs, depression, presence of comorbidities, and post-traumatic stress symptoms. Clinicians and other health care providers may focus on preventing persistent pain and shifting the focus towards prevention of chronicity following an injury.

## Figures and Tables

**Table 1:** Study characteristics.

Studies	Year of Publication	Country of Study	Study Design	Participants	Follow-upPeriods	Outcome	Risk Factors	Sample Size
Hallegraeff et al. [27]	2020	The Netherlands	Longitudinal prospective cohort	Acute low back pain (LBP with <6 weeks duration with or without radiating pain and had been pain free for at least 3 months before the onset of their current back pain) Age 18–60 years	Baseline and 12 weeks	Pain: NRS and PDI Anxiety: STAI-Y (STAI-S and STAI_T)	Pain intensity at outset Duration of pain Physical workload State and Trait Anxiety	225
Akerblom et al. [28]	2019	Sweden	Retrospective cohort	Persistent pain following neck trauma	NR	Anxiety and depression: HADS Acceptance: CPAQ-8 Pain: distribution and severity: MPI	Participant demographics, anxiety, depression, acceptance, persistent pain	565
Modarresi et al. [23]	2019	Canada	Retrospective Exploratory cohort	Adults > 18 years who were recovering from distal radius fracture	Baseline, 3, 6, and 12 months	Pain and disability: PRWE Comorbidities: SCQ	Depression, participant demographics, education and employment status, pain and disability	318
Friedman et al. [19]	2018	USA	Retrospective cohort	Acute LBP	Baseline, 1 week following ED visit and 3 month follow-up	LBP-related functional impairment (RMDQ) Presence of moderate or severe LBP	Pain and functional impairment	354
Soderlund et al. [29]	2018	Sweden	Prospective cohort	General population with whiplash history 2–4 months prior to recruitment. WAD grade 1–2 Age 18–65 years	Baseline, prior to discharge and 1 year follow-up	CPAQ MPQ TSK	Pain acceptance, fear of movement and fear of (re)injury	177
Wellsandt et al. [21]	2018	USA	Prospective cohort	Athletes with acute, unilateral ACL injury Athletes were level 1 or 2 athletes	5 years post initial injury: Baseline), immediately following 10 additional physical therapy sessions and 6 months following completion	Quadriceps strength: MVIC Impairment and functional limitation KOS-ADLS General function GRS IKDC	Knee function	66
Silva et al. [20]	2018	USA	Case-control study	Student or professional musicians with upper limb injuries Age 18–65 years	NR	Cervical flexor endurance test Scapular dyskinesis test Craniocervical test	Motor control	72
Andersen et al. [24]	2016	Denmark	Longitudinal cohort	General population admitted to hospital emergency department with traumatic whiplash QTFC-WAD grade 1–3 Age > 18 years	Baseline, 3 months and 6 months	Pain: NRS Fear-avoidance beliefs: Orebro musculoskeletal pain screening questionnaire PTSS: Harvard trauma questionnaire Depressive symptoms: HADS Catastrophizing: PCS	Pain at outset Pain catastrophizing PTSS Depression Fear avoidance	198
Heidari et al. [25]	2016	Germany	Longitudinal cohort	Presence of non-specific back pain Participation in some form of active exercise therapy Age > 18 years	6 months	Back pain: Chronic pain grade Stress: Recovery stress questionnaire and trier inventory for assessment of chronic stress	Pain and chronification stress	139
Rosenbloom et al. [22]	2016	Canada	prospective, observational, longitudinal design	Traumatic musculoskeletal injury Admitted to hospital for >2 days Age > 18 years	14 days and 4 months	Neuropathic pain: self-report Leeds assessment of neuropathic symptoms and signs and NRS BPI Mental health: HADS Pain anxiety: pain anxiety symptom scale Post-traumatic stress disorder checklist pain self-efficacy checklist Pain catastrophizing scale Anxiety sensitivity index	Chronicity	128
Pierik et al. [26]	2016	The Netherlands	Prospective 1 year follow-up study	Isolated musculoskeletal injury caused by blunt trauma Age 18–69 years	1 week, 6 weeks, 3 months, and 6 months	Pain: NRS HRQoL: SF-36 Anxiety and depression: HADS Pain Catastrophizing: PCS Kinesiophobia: TSK Pain experience during follow-up: BPI	Chronic pain 6 months post-injury	435
Holmes et al. [16]	2013	Australia	3 year follow-up cohort	Scored >2 on abbreviated injury score Admitted for more than 24 h Age 18–70 years	3 months, 12 months, and 3 years	Pain: NRS Disability: SF-36 Social Support: Multidimensional scale of perceived social support Mental health: NRS Psychological symptoms: HADS	Presence of chronic pain Pain-related disability	220
O’Connor et al. [32]	2013	United Kingdom	Secondary analysis	Acute ankle injury Age > 16 years	4 weeks and 4 months	Pain: Y/N Injury grade	Ankle function	85
Holmes et al. [17]	2010	Australia	prospective cohort with 12 months follow-up	Scored > 2 on abbreviated injury score Admitted for more than 24 h Age 18–70 years	3 months and 12 months	Pain: NRS Disability: SF-36 Social Support: Multidimensional scale of perceived social support Mental health: NRS Psychological symptoms: HADS	Presence of chronic pain Pain-related disability	238
Williamson et al. [18]	2009	Australia	Prospective cohort study	Admitted to hospital with orthopedic injury	In hospital and 6 months	SF12 Pain: VAS	Chronic pain 6 months post-injury	1290
Harris et al. [15]	2007	Australia	Cross- sectional study	Major trauma after accidental injury Age > 18 years	1–6 years post-injury	PTSD: PTSD checklist Back pain in the preceding week General health: SF-36 Disability: ODI	NR	355
Kovacs et al. [30]	2005	Spain	Longitudinal study	Acute LBP with or without radiation to leg	14 days, 59 days	Pain: VAS Disability: RMQ and EQ-5D	Pain and disability	366
Potter et al. [31]	2000	United Kingdom	Prospective longitudinal study	Uncomplicated musculoskeletal pain Age 18–65 years	Baseline and 12 weeks	Health: general health questionnaire Pain: VAS, pain measurement inventory Coping: active coping score and passive coping score	chronicity	141

**Table 2:** Quality scores from the 18 included studies.

Factor	Hallegraeff et al. (2020) [27]	Akerblom et al. (2019) [28]	Modarresi et al. (2019) [23]	Friedman et al. (2018) [33]	Soderlund et al. (2018) [29]	Wellsandt et al. (2018) [21]	Silva et al. (2018) [20]	Andersen et al. (2016) [24]	Heidari et al. (2016) [25]	Rosenbloom et al. (2016) [22]	Pierik et al. (2015) [26]	Holmes et al. (2013) [16]	O’Connor et al. (2013) [32]	Holmes et al. (2010) [17]	Williamson et al. (2009) [18]	Harris et al. (2007) [15]	Kovacs et al. (2005) [30]	Potter et al. (2000) [31]
Study participation summary	L	L	L	L	L	L	L	L	H	L	H	L	L	L	L	L	L	L
Study attrition summary	L	M	M	M	L	L	L	H	H	L	M	M	M	M	L	H	H	H
Prognostic factor measurement summary	L	L	M	M	L	M	H	L	L	L	L	L	M	L	M	L	L	H
Outcome measurement summary	L	L	L	L	L	L	L	L	L	L	L	L	L	L	M	L	L	H
Study confounding summary	L	L	M	M	L	M	H	L	H	L	L	L	H	L	H	H	H	H
Statistical analysis and presentation summary	L	L	L	L	L	L	L	L	L	L	L	L	L	L	L	M	L	H
Overall	+++	+++	++	++	+++	++	+	++	+	+++	++	+++	++	+++	++	+	+	+

H: High bias; M: Medium Bias; and L: Low Bias. High quality (+++): Majority of criteria met, little or no risk of bias. Results unlikely to be changed by further research. Acceptable (++): Most criteria met. Some flaws in the study with an associated risk of bias, Conclusions may change in the light of further studies. Low quality (+): Either most criteria not met, or significant flaws relating to key aspects of study design.

**Table 3:** Risk factors for causing persistent pain following musculoskeletal injuries.

Citations	Age, Y Mean (SD)	Gender, n (%)	Activity	Injury Type	Region	Risk Factors	Results
Hallegraeff et al. [27]	41 (12)	Female 103 (51%)	Physically active 141 (69%)	Non-specific acute LBP	Lumbar	State and trait anxiety Pain intensity at outset Pain related disability Duration of LBP Widespread pain	State anxiety levels (OR 1.1 (95% CI 1.0–1.1, *p* = 0.00)) and pain intensity (OR 1.3 (95% CI 1.1–1.7 *p* = 0.01)) at baseline were independent predictors of still having pain at 12 weeks. Trait anxiety was not found to be predictive of pain at 12 weeks.
Akerblom et al. [28]	Median age 39			Traumatic neck injury	Neck	Participant demographics, anxiety, depression, acceptance, persistent pain	Widespread Pain: females and lower acceptance Pain Interference: females, depression, and lower acceptance Pain Severity: lower acceptance, increased levels of anxiety or depression, and lower education level.
Modarresi et al. [23]	59.6 ± 11.9	Female 80.5%	NR	Distal radius fracture	Wrist	Depression, participant demographics, education and employment status, pain and disability	Majority recovered within normal limits, depression was associated with non-recovery 24% v 8%, X^2^ = 6.36, *p* = 0.01 (rapid recovery) and 16%, X^2^ = 4.07, *p* = 0.04 (slow recovery) No other factors associated with slow/non recovery.
Friedman et al. [33]	38 (12)	Female 160 (45)	NR	Acute low back pain	Lumbar	Pain one week following injury Functional impairment	At the 3 month follow-up 39% of patients reported LBP related functional impairment and 16% reported moderate to severe LBP. The baseline STaRT score was not associated with long-term pain. The length of pain duration anticipated by the patient (>7 days) was associated with both the pain at 3 months (OR 2.31 (95% CI 1.17–4.54)) and functional disability (OR 1.93 (95% CI 1.09–3.43))
Soderlund et al. [29]	39.5	Female 225	NR	Whiplash	Neck	Fear of movement and fear of (re)injury Pain acceptance	Patients with support from significant others and lower levels of fear of movement and better outcome predictions were associated with better outcomes at the 1 year follow-up than those without.
Wellsandt et al. [21]	Non-OA 28.8 (11.3) OA 28.3 (11.5)	Non-OA Male/ Female: 43/24 OA Male/ Female: 6/3	Level 1 52 Level 2 24	ACL	Knee	Knee function	The risk of developing knee OA 5 years after experiencing an ACL injury is increased when individuals had poor performance in the single-legged hop test. This result was not the same as patients who underwent ACL reconstruction.
Silva et al. [20]	Symptomatic 23.3 ± 8.21 CG 25.03 ± 10.5	EG: M/F 12/24 CG: M/F 12/24	Exercise Days 3.1 Minutes 164.9	Upper limb and neck pain	Upper limb and neck	Motor control	Musicians who present with upper quadrant playing-related pain had reduced performance in clinical tests and demonstrated poor scapular motor function.
Andersen et al. [24]	36.79 (12.61)	Female, 61.6%	NR	Whiplash injury	Neck	Demographics Fear avoidance (FA) beliefs Catastrophizing Depression	35.4% as non-recovered. The non-recovered (the medium stable, high stable and very high stable trajectories) displayed significantly higher levels of post-traumatic stress symptoms (PTSS), pain-catastrophizing (PCS), FA, and depression compared to the recovered trajectories. Importantly, PCS and FA beliefs mediated the effect of PTSS on pain intensity
Heidari et al. [25]	32.24 (11.32)	Female, 41%	Athletes	Musculoskeletal pain	Back	Pain factors Stress	No significant differences noted between the chronic group and non-chronic group, insignificantly elevated stress levels.
Rosenbloom et al. [22]	43.0 (19.9)	Female, 32.2%	NR	Motor- vehicle accidents	Multiple locations	Demographics, Pain factors, Mental health	The deleterious effects of neuropathic pain were seen in the 32% of young trauma patients who had symptoms of neuropathic pain 4 months after injury. The pain interfered significantly with their daily living, employment, mood, sleep, and enjoyment of life.
Pierik et al. [26]	Median: 50.0 (IQR 36.0–60.0)	Female, 60.5%	NR	Fracture: 328 (75.4%) Dislocation: 25 (5.7%) Sprains and Strains: 47 (10.8%) Contusion: 24 (5.5%) Muscle rupture: 10 (2.3%)	Lower extremity	Demographics Pain factors Psychological factors Injury and treatment factors Clinical Factors	Age: 40–49: OR 1.03 (95% CI 0.28–1.07); 50–59: OR 3.43 (95% CI 1.29–9.09); 60–69: OR 3.85 (95% CI 1.47–10.08) Pain level at discharge, severe pain: OR 3.41 (95% CI 1.73–6.71); Preexisting chronic pain: OR 6.09 (95% CI 3.18–11.69); Pre-injury physical, Poor: OR 3.18 (95% CI 1.68–6.02); Comorbidities, yes: OR 2.87 (95% CI 1.53–5.40)
Holmes et al. [16]	Chronic Pain: 41.4 (13.0) No Chronic pain: 38.5 (13.1)	Chronic Pain, female: 31% No Chronic pain, female: 27%	NR	Multiple trauma	Multiple locations	Demographics Pain factors Psychological factors Social support	Initial pain: OR 1.26 (95% CI 1.09–1.46); Injury severity: OR 1.12 (95% CI 1.01–1.24)
O’Connor et al. [32]	27 (9.8)	Female, 30%	NR	Inversion sprain	Ankle	Demographics Injury variables	Increased risk of poor function pain med joint line: 4.92 (95% CI 1.39–8.44); pain weight-bearing ankle dorsiflexion: 6.8 (95% CI 4.8–8.7)
Holmes et al. [17]	Chronic Pain: 42 (14) No Chronic pain: 39 (14)	Chronic Pain, female: 71% No Chronic pain, female: 75%	NR	Multiple trauma	Multiple locations	Demographics Pain factors Psychological factors Social support	Number of injuries: OR 1.14 (95% CI 1.02–1.27); Initial pain: OR 1.34 (95% CI 1.13–1.61); Pain control attitudes: OR 0.79 (95% CI 0.69–0.99)
Williamson et al. [18]	Range: 14–95	Female: 39%	NR	Multiple trauma	Multiple locations	Demographics, Pain factors Function	Self-reported pre-injury, pain-related disability, and moderate or severe pain at discharge from the acute hospital were found to be independent predictors of moderate or severe pain at 6 months post-injury.
Harris et al. [15]	47.8 (19–91)	Female, 28%	NR	Musculoskeletal pain	Back	Demographic, Clinical factors Injury severity Psychosocial factors	PTSD: OR 4.92 (95% CI 2.83–8.56); >3 chronic illness: OR 5.83 (95% CI 2.41–14.09). The presence of back pain was significantly associated with increasing chronic illnesses at follow-up.
Kovacs et al. [30]	47.7 (15.5)	Female, 54%	NR	Musculoskeletal pain	Low back	Demographics, Pain factors Function	The more pain an individual had at baseline the increased risk of disability at 60 days follow-up.
Potter et al. [31]	Chronic Pain: <40 = 28 (41.2%) 40–50 = 23 (33.8%) >55 = 17 (25.0%) Acute Pain: <40 = 36 (49.3%) 40–50 = 15 (20.5%) >55 = 22(30.1%)	Chronic Pain, female: 64.5% Acute Pain, female: 53.4%	NR	Musculoskeletal pain	Multiple locations	Demographics, Health status Pain factors	Pain intensity, active coping score, and previous episode of continuous pain were significantly and independently related to the development of chronic pain.

## Data Availability

Not applicable.
